# Role of the Microbiota in Lung Cancer: Insights on Prevention and Treatment

**DOI:** 10.3390/ijms23116138

**Published:** 2022-05-30

**Authors:** Federica Pizzo, Zaira Maroccia, Ivano Hammarberg Ferri, Carla Fiorentini

**Affiliations:** 1 Association for Research on Integrative Oncology Therapies (ARTOI), Via Ludovico Micara, 73, 00165 Rome, Italy; pizzo.fede@gmail.com (F.P.); ivanoferri@yahoo.com (I.H.F.); 2 Department of Cardiovascular, Endocrine-Metabolic Diseases and Aging, Istituto Superiore di Sanità, Viale Regina Elena, 299, 00161 Rome, Italy; zaira.maroccia@iss.it

**Keywords:** lung neoplasms, lung neoplasms, prevention and control, lung neoplasms, therapy, carcinoma, non-small-cell lung, gut–lung axis, lung microbiota, gut microbiota, host–microbial interactions, mucosal tissue, immune system

## Abstract

The microbiota is increasingly recognized as a critical player in cancer onset and progression and response to cancer chemotherapy treatment. In recent years, several preclinical and clinical studies have evidenced the involvement of microbiota in lung cancer, one of the world’s deadliest cancers. However, the mechanisms by which the microbiota can impact this type of cancer and patient survival and response to treatments remain poorly investigated. In this review, the peculiarities of the gut and lung microbial ecosystems have been highlighted, and recent findings illustrating the possible mechanisms underlying the microbiota–lung cancer interaction and the host immune response have been discussed. In addition, the mucosal immune system has been identified as a crucial communication frame to ease interactive dynamics between the immune system and the microbiota. Finally, the use of specific next-generation intestinal probiotic strains in counteracting airway diseases has been evaluated. We believe that restoring homeostasis and the balance of bacterial microflora should become part of the routine of integrated cancer interventions, using probiotics, prebiotics, and postbiotics, and promoting a healthy diet and lifestyle.

## 1. An Introduction to Gut and Lung Microbiota

More than 100,000 trillion microorganisms entirely colonize the human body, constituting the microbiota, a microbial system resident in and on the human body, with a crucial role in host health maintenance [1,2].

Microbial communities are as unique as fingerprints and vary in the same individual during the lifespan and with diet or drug treatments [3,4]. The microbiota co-evolves, modulating all the physiological processes of the host, from conception to death, influencing also subsequent generations throughout the maternal microbiota [5,6], strongly regulating multiple host functions, including metabolic processes, nutritional responses, circadian rhythmicity [7], and playing a crucial role in immune system development and training [8].

Obtaining a definition of a “healthy microbiome” is highly complex due to the functional redundancy of microbial species; hence, different taxonomic profiles can lead to microbial communities with similar behaviors [6]. However, a microbial ecosystem in stable equilibrium, characterized by a preponderance of potentially beneficial species in harmonic relationship with each other, is defined as a state of eubiosis [9]. Accordingly, when an imbalance between species occurs, the beneficial species/pathogenic relationship is inverted and leads to a status of dysbiosis.

Indeed, the intestinal mucosa is the area in charge of contact and interaction with the external environment, characterized by the most incredible variety and density of microorganisms. The intestinal microbiota is the most studied and best treated microbial ecosystem, a leader in controlling physical and mental health status, and a major player in the gut–brain axis [10,11]. Given that a lot of information is available on the gut microflora, a field that deserves deeper investigation is the one focused on the lung microbiota. 

Although the lungs have a large mucosal surface exposed to microorganisms due to contact with the external environment, the bacterial biomass detected in healthy lungs appears to be very low (5–8.25 log copies/mL). It was found that more than 90% of the microbiome detected with next-generation sequencing could correspond to nonviable bacteria [12]. This finding agrees well with the low bacterial density highlighted in the lung area, probably due to the robust immune response of the pulmonary system mainly mediated by macrophages, the capacity for active elimination of microorganisms by the pulmonary epithelium, and the inhospitable conditions of the respiratory tract for bacterial life. Overall, the lung microbiota is a highly dynamic system whose equilibrium is maintained by continuous microbial integration and elimination and by the homeostasis of regional growth conditions, such as oxygen tension, temperature, pH, availability of nutrients, cell activation, inflammation, and local microbial competition [13].

Several studies have identified many different “pneumotypes” in individuals [14,15,16]. For example, Segal et al. observed that a bacteriome enriched in oral taxa (*Prevotella* and *Veillonella*) was related to the Th17-dependent local inflammatory response, which appears crucial in modulating lung immune status, both in healthy and pathological conditions [14]. Although the lung microbiota is recognized as a milestone in the pathophysiology of several respiratory diseases, a clear connection between the composition of the lung microbial community and homeostasis is still not defined and certainly deserves further and deeper investigations.

## 2. Gut–Lung Axis (GLA)

The gut and the lungs have the same embryonic origin [17] and have physical interactions, since ingested microorganisms can access both gastrointestinal and respiratory tracts, and the gastroesophageal contents can enter the lungs through inhalation [18]. Thanks to this extensive dialog between the gut and the lungs, the GLA has, in recent years, emerged to have a notable role in health and disease [19]. 

As with all organs belonging to the compartments of the mucosal immune system (MIS), the gut and the lungs are covered by a similar mucosa, hence favoring the same dynamics in the interactions between the immune system and their microbiota [20]. Furthermore, they are indirectly linked through the lymphatic and circulatory systems, so the stimuli that locally impact the immune system have also systemic effects, whereas the intensity of the immune response depends on the site of the first interaction [21]. The affinity between the two organs also concerns the microbial communities, since the predominant bacterial phyla in healthy subjects’ lungs, i.e., *Firmicutes* and *Bacteroidetes*, are the same as in the intestine [12,22,23]. However, the effect of the intestinal microbiota on the lungs and other systems is not clear and the influence of the lung microbial community on the intestine is still poorly explored [24].

An increasing number of studies identify MIS as the mechanistic basis of the gut–lung interaction, by means of the stimulation of the immune system cells performed by the microbial communities (T and Treg cells, B cells, major histocompatibility complex, dendritic cells, intestinal epithelial cells), by the synthesis of specific secretory IgA and antimicrobial peptides (AMPs) [25,26], and by the production of certain microbial metabolites, such as tryptophan catabolites and short-chain fatty acids (SCFAs) [24,27].

The causal link between intestinal dysbiosis and the development of asthma, lung infections, and other respiratory disorders has already been proven in in vivo research. Recently, Brown et al. [28] have shown that the intestinal microbiota promotes resistance to respiratory infections by inducing the production of IL-17A in the gut, which in turn increases the production of the granulocyte-macrophage colony-stimulating factor in the lung and activates the process of killing and clearance of pathogens by macrophages, at the alveolar level.

The lung microbiota can also affect intestinal immunity since it has been observed that *Staphylococcus aureus* or *Pseudomonas aeruginosa* pneumonia can induce sepsis and apoptosis processes in the gut [29].

IgA, the first defense of the mucosal barrier against microbial antigens, is the MIS predominant antibody isotype. About 80% of the body’s plasma cells (PCs) are found in the gut mucosa, where the greatest production of IgA is detected [30]. They are also present in tears, saliva, and colostrum, as well as in the gastrointestinal, respiratory, and vaginal tracts [31]. Although the underlying mechanisms are still unclear, IgA is the main actor in the balance between immune protection and tolerance and decisively contributes to maintaining mucosal homeostasis and implementing non-inflammatory response strategies [30]. Regarding the role in the balance between protection and tolerance, the treatment of intestinal infections with certain probiotics (*Lactobacillus casei*, *Lactobacillus rhamnosus*, *Bifidobacterium bifidum*, and *Bifidobacterium infantis*) significantly increases the number of pathogen-specific sIgA, while the production of probiotic-specific sIgA was not induced, indicating the distinction from pathogens and tolerance towards beneficial bacteria [32]. This class of immunoglobulins regulates the density of the microbial community through low-affinity connections with microorganisms or parts of them and, at the same time, neutralizes toxins and pathogens by means of high-affinity bonds. Therefore, IgA can lead to both innate and adaptive immune responses [26]. The main site of IgA induction is the gut-associated lymphoid tissue, or rather in Peyer’s patches (PPs), in isolated lymphoid follicles and mesenteric lymph nodes. Nasopharynx-associated lymphoid tissues and bronchial-associated lymphoid tissues are also IgA inductive MIS sites. Mucosal antigens reach PPs via the transcytosis of M cells or are captured directly by dendritic cells (DCs), which present the antigen by stimulating B and T cells to produce specific IgA [31]. After terminal differentiation into PCs, IgA + B cells circulate through the lymph and bloodstream and spread IgA-secreting cells to reach other effector sites, whereby IgA cells are responsible for local and systemic responses [26].

Another efficient tool of innate mucosal immunity is represented by AMPs produced by Paneth’s epithelial cells, capable of modeling the intestinal microbiota composition and whose barrier action is essential for survival. A recent study has shown high mortality due to systemic microbial translocation and inflammation in mice unable to produce adequate amounts of AMP [33].

SCFAs, derived from the fermentation of dietary fibers by intestinal bacteria, are key mediators of the host–microbiome interaction and perform countless functions with localized and systemic effects. The basic function of these fatty acids is to provide energy, and it has been calculated that they provide the whole organism with 10% of the daily energy needed [34] and with the 70% of the energy needed by enterocytes [35]. SCFAs also act as signaling molecules by mediating metabolic processes and immune responses, and various studies have proven their impressive anti-inflammatory action and antitumoral potential. Davie [36] observed that butyrate could inhibit the activity of histone deacetylase by epigenetic regulation of the expression of genes responsible for cell proliferation or apoptosis, such as the p21 gene (Waf1/Cip1). SCFAs can also activate G protein-coupled receptors (GPCRs) expressed by epithelial enteroendocrine cells, polymorphonuclear hematopoietic cells, and smooth muscle cells, thus regulating their functions. For example, butyrate has been shown to promote anti-inflammatory processes by activating the colon GPCR GPR109A, which in turn induces the production of anti-inflammatory interleukins IL-10 and IL-18 by macrophages and DCs [37]. Furthermore, it has been observed that through the binding to the GPR41 receptor of innate lymphoid cells and CD4 + T cells, SCFAs lead to the production of IL-22, a cytokine with an anti-inflammatory action crucial for the homeostasis of the epithelial barrier, which also stimulates the expression of antimicrobial peptides and mucin secretion [38].

Moreover, the tryptophan catabolites produced by the bacteria of the microbiota stimulate the production of IL-22 following binding to the abundance of aryl hydrocarbon receptors present on the mucosal surfaces, contributing to their protection [39]. Several fundamental studies have focused on the beneficial effect of the microbial metabolites produced in the intestine on the mucous membranes of distant sites, such as the lung. Trompette and coworkers [40], for example, have seen that in mice fed a high-fiber diet, circulating SCFA levels increased and allergic inflammation in the lungs decreased. On the contrary, inflammation increased with decreasing levels of SCFAs, demonstrating how the latter can shape the immunological environment in the lung. Moreover, Cait and coworkers [41] showed how the SCFAs produced in the intestine act in the lungs by modulating the activity of T cells and resident DCs, improving the asthmatic condition in mice with dysbiosis. Although immune regulation has been well studied in isolated mucosal sites, the immune interaction between anatomically distant mucosal sites is far from being understood. Considering this, a holistic and integrated approach to the study of the MIS is critical for future advances in understanding mucosal immunology, particularly for the treatment of cancer and other chronic diseases [42].

## 3. Microbiota and Cancer in Mucosal Tissues

A number of studies show how the microbiota influences the onset and development of cancer, impacting the processes of tumoral transformation and progression and the response to anticancer therapies [43]. In fact, it is widely known that bacteria can modulate immune responses, inflammatory status, metabolism, and cell proliferation, and to mediate epigenetic and genotypic processes [44]. The effect of the intestinal microbiota in regulating tumors in the gastrointestinal tract and in the distal sites is a widely investigated field. The microbiota of tumor-bearing tissue also forms a crucial part of the tumor microenvironment, influencing local tumorigenesis and tumor progression [45].

Defining the close relationship between microbiota, cancer, and mucous tissues is a challenging field of study. Today, the immune system could be considered the “fil rouge” that connects these different entities, especially regarding the innate immunity sector, which plays a crucial role in the immune surveillance of cancer and in the functionality of the MIS. 

Specifically, the mucosal-associated invariant T (MAITs) cells are a population of mucosal innate immunity T-like cells that recognizes specific bacterial metabolites presented by molecules with the MR1 antigen [46]. The acknowledgment of the potential of MAITs has rendered them extremely interesting in the development of therapies for diseases such as HIV, tuberculosis, and cancer [47]. The activated MAIT cells are highly proliferative and perform a cytolytic action mediated by granzyme B and perforin towards target cells. In addition to the antigenic recognition of a wide range of bacteria, they can also be recalled and activated by chemokines [48]. MAIT cells have been found to be particularly active in the control of intracellular infections, as seen in *Mycobacteria tuberculosis* lung infections [49], and this could be a very interesting aspect when related to the observation of Nejman and collaborators [45] regarding the intracellular localization of intratumoral bacteria. The role of MAIT cells in tumor immunity is still debated. In 2016, the activity of MAIT cells was linked with the development of tumors of the gastric, lung, and colon mucosa [50]. Precisely, MAITs migrated massively from the circulation to the tumor site of the colon due to chemokine recall. However, despite the antitumor potential shown in vitro in K562 cells, their peripheral circulatory deficiency and their presence in the tumor site have been correlated with progression and a worse prognosis for mucosal-associated tumors.

Although the modeling and regulating functions of the intestinal microbiota on the host’s immune system are recognized, the communication methods and movements of immune cells between the intestine and other organs need further investigation. Starting from the study of the anatomical and functional distribution mechanisms of natural killer (NK) cells, which are fundamental in response to viral infections and in cancer immunosurveillance, distinguished into the main categories of immature/precursor and differentiated/effector, it has been observed that immature and precursor NKs were predominantly present in lymph nodes and intestines in a tissue-specific manner, functioning as organism reservoir sites [51]. The differentiation triggered the distribution via the bloodstream to the bone marrow, spleen, and lungs, where they performed immunosurveillance to exert their cytotoxic action. The same authors highlighted that the preferential distribution of effector NKs was consistent with their role in identifying infected and metastatic cells.

A study conducted in 2014 [52] focused on transgenic mice for a photoconvertible fluorescent protein. Impressive lymphocyte traffic to and from the intestine and the proximal and distal lymph nodes was recorded, and attention was paid to the movements of Th17 cells, notoriously involved in the distal responses most influenced by the gut. The central role of the intestinal environment in the formation of immune responses has been confirmed again.

Much attention should also be paid to B cells and tumor-infiltrating PCs, whose presence been associated with better prognosis in ovarian cancer [53]. Biswas and coworkers showed instead how the IgA-mediated humoral response regulates immunity against ovarian cancer and is associated with a better prognosis. In detail, it was found that the IgA not specific for tumor antigens, produced by the infiltrating B cells, redirected the T and B cells themselves against the tumor, causing cytolysis. In addition, thanks to the activation of internal transcriptional changes, the process of transcytosis of IgA through the tumor cells makes them sensitive to attack by T lymphocytes [54].

Another particularly intriguing target is represented by extracellular vesicles produced by intestinal immune and epithelial cells and by commensal bacteria. The content of the exosomes, which varies according to the environment and the type of message to be communicated, is generally referred to as a set of bioactive effectors and genetic material (e.g., microRNAs, antigens, AMP, growth factors, etc.) [55]. Such vesicles have been shown to be involved in innumerable physiological and pathological processes. In an interesting and complex study, it was observed in vitro and ex vivo that microvesicles produced by the probiotic *L. rhamnosus* JB-1 induced DCs towards an immunoregulatory phenotype and even influenced the peristaltic activity by acting directly on the enteric nerve [56]. Again, Zhang and coworkers showed that exosomal veins produced by gastric carcinoma cells transported and translocated the EGFR receptor in liver cell membranes, triggering the metastatic process in the liver [57]. Another key interaction of extracellular vesicles concerns NK cells, which can be activated or suppressed by exosomes produced by DCs, cells subjected to inflammatory stress by tumor cells [58,59].

## 4. Microbiota in Lung Cancer 

**Figure 1:**
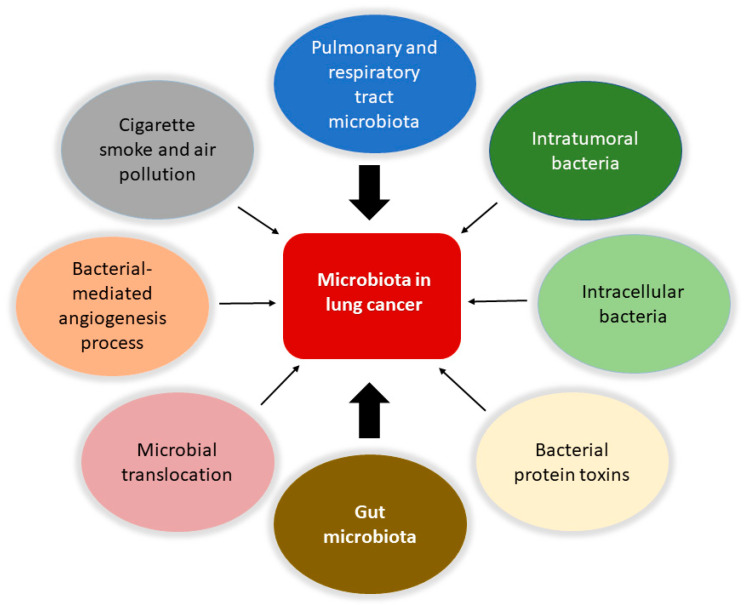
Involvement of the microbiota in lung cancer. The microbiota is involved in the biology of lung cancer at different levels and through various mechanisms. Protagonists are the bacteria in the upper respiratory and pulmonary tract and those that constitute the tumor microenvironment with intratumoral and intracellular localization. The intestinal microbiota plays a central role in modulating the responses of the immune system and the inflammatory state of the body, together with the bacteria involved in translocation phenomena in the bloodstream. It is also possible that certain bacterial toxins that can activate oncogenic pathways may lead to transformation in the lung. Furthermore, pollution and cigarette smoke are directly responsible for dysbiotic changes in the lung. Finally, the microbiota can affect metastasis processes by increasing the expression of vascular endothelial growth factors and promoting inflammation.

Accordingly to recent estimates, lung cancer is the principal cause of mortality due to cancer and the second most common cancer type [17,60]. It is evaluated that 90% of all lung cancer cases can be attributed to smoking, with tobacco smoke, air pollution, and other carcinogens established risk factors. However, the exact mechanisms are not well understood. As the mucosa site with the largest surface area in the body and an essential interface with the external environment, the lung represents a unique opportunity for exposure to microbes and environmental challenges. Although traditionally thought to be sterile, the lung is home to a wide range of microbes. The prevalence of microbes is dictated by the immigration of new bacteria, mechanical and immune elimination, and replicative success in local environmental conditions. The lung microbiota appears to be dysregulated in lung diseases such as chronic obstructive pulmonary disease and cystic fibrosis [13,61,62,63], and cancer (Figure 1).

Compared to the gastrointestinal tract, the lung microbiota is poorly understood. A study investigating female, never-smoker, lung cancer patients, demonstrated a correlation between gut microbiota and TNM stage and primary tumor size [64]. In detail, there is a significant positive correlation between the relative abundance of *Faecalibacterium* and T category (TNM) and primary tumor size, and a negative correlation between the relative abundances of *Fusicatenibacter saccharivorans* and *Bacteroides* and T category and primary tumor size. EGFR WT (wild-type) seems to be correlated to a higher relative abundance of *Bifidobacterium* and *Faecalibacterium* and a lower relative abundance of *Blautia*, compared to EGFR mutated patients. It is currently impossible to establish whether these gut microbiota changes are prior to cancer development or follow the disease. It is worth remembering that the effect of these changes is potentially due to the influence on the immune system by bacterial biochemical metabolites or molecules, as is the case of *Bacteroides* that can upregulate T cells in the tumor microenvironment, suppressing tumor proliferation, or the case of *Faecalibacterium,* that can activate T regulatory cells playing a role in cancer progression. 

It has also been reported that Gram-negative bacteria, such as *Haemophilus influenza*, *Enterobacter spp*., and *Escherichia coli*, tend to colonize lung cancer. Regarding the gut microbiota of lung cancer patients, when compared to healthy individuals, it displays a lower concentration of *Firmicutes* and *Proteobacteria*, combined with relatively higher levels of *Bacteroidetes* and *Fusobacteria* [65]. These phyla were found constantly, regardless of microbial changes in cancer. 

Furthermore, when the intestinal microbiota and its products are translocated through the epithelial barrier and then into the blood flow, they stimulate a toll-like receptor (TLR) response and the subsequent expansion of T lymphocytes into distant tissues. The translocation of bacteria from the gastrointestinal tract can enhance tumor-specific responses through TLRs or the induction of memory responses, as observed for the relationships between *Enterococcus hirae* and small-cell lung cancer [66,67].

Increasing evidence indicates that the conversion of the gut microbiota from a mutualistic configuration into a pro-carcinogenic configuration may be favored by triggering factors that comprise inflammation and bacterial infections. Several bacterial pathogens can produce enzymatically active protein toxins that can directly attack and damage DNA or become involved in essential host cell signaling pathways that direct cell proliferation, apoptosis, and inflammation [68]. The *E. coli* colibactin and cytolethal distending toxin (CDT) are significant examples of bacterial toxins able to induce mutations and genome instability, whereas the *Bacteroides fragilis* toxin, the *E. coli* Cif and cytotoxic necrotizing factor 1 (CNF1), the *Fusobacterium nucleatum* FadA, and the *Salmonella* AvrA are prototypes of toxins that engage signaling pathways, ultimately leading to transformation [68,69]. Chronic inflammation can contribute to colorectal cancer via several mechanisms, including the induction of the epithelial–mesenchymal transition (EMT), a process involved in metastasis, invasion, and cancer progression. CNF1 and FadA have been reported to trigger the EMT, and also the protein toxins CagA and CagE, from the pro-carcinogenic bacteria *Helicobacter pylori*, which raises the patients’ risk for gastric cancer [70]. Interestingly, *H. pylori* infection has also been associated with lung cancer since its inhalation may lead to lung tissue colonization that can cause direct damage and chronic inflammation. One of the *H. pylori* protein toxins, VacA, exerts a cytotoxic effect in airway epithelial cells and triggers the production and secretion of the pro-inflammatory cytokines Il-6 and IL-8 [71]. Hence, in concomitance with environmental risk factors and genetic predispositions of the host, *H. pylori* and its toxins could be involved in lung cancer onset [72]. 

Knowing the lung microbiota composition could represent a valuable tool for prognostic investigation. In fact, in early-stage lung cancer patients, the gut microbiota undergoes characteristic changes that permit the identification of these possible bacterial contributors to lung cancer development [73]. In particular, controls show a higher abundance of the genus *Bifidobacterium* and *Faecalibacterium*, whereas *Bacillus* spp. appears more elevated in lung cancer patients. *Bacillus* spp. detected in lung sputum have been connected to an increased lung cancer risk [74]. The dysbiosis of gut and sputum microbiota is associated with disease progression and distant metastasis (DM), but the sputum microbiota is best for discriminating stage I to III patients from DM patients [75]. Lu and coworkers observed a progressive worsening in the alpha diversity of sputum microbiota when comparing stage I to stage III patients and to DM patients. The *Coriobacteriaceae* family and the genera *Actinomyces, Streptococcus*, and *Pseudomonas* are significantly increased while *Capnocytophaga* was decreased in the sputum and the gut microbiota of DM patients. A correlation was found between brain metastasis (BM) and *Pseudomonas* while trying to identify potential microbial biomarkers. There was no detectable pseudomonas in the other stages of lung cancer or healthy controls, while it was present in the fecal microbiota of BM patients and highly abundant in the sputum; thus, there is a significant association between pseudomonas and BM in non-small-cell lung cancer (NSCLC).

Distant modulation by the gut microbiota of the immune system, cancer progression, and metastasis growth are well explained in a mouse model of lung metastatic melanoma [76]. The supplementation of specific strains of *Lactobacillus* and *Bifidobacterium*, increases the gut microbiota relative abundances of *Lachnospiraceae*, *Streptococcus*, and *Lachnoclostridium*, which are all involved in SCFAs production. In the presence of a sufficient intake of fiber, the concentration of SCFAs in the gut increases and thus in the blood, as well as in the sputum, indicating that they reach the lungs and the airways. The distant effect of SCFAs promotes the expression of CCL20 (also known as LARC or MIP3A) in lung endothelial cells, which in turn recruits Th17 cells that attenuate melanoma cell metastasis in the lung. Moreover, it has been observed that microorganisms are closely related to tumor angiogenesis and metastatic processes by regulating the expression of the vascular endothelial growth factor (VEGF) and inflammation. Wang and collaborators highlighted the ability of *H. influenzae*, *Human papillomavirus*, and *H. pylori* to increase the expression of angiogenic mediators, chemokines, and cytokines that promote angiogenesis and inflammation in lung cancer [77]. In contrast, the fungi zj-14, zj-17, and zj-36, *Akkermansia muciniphila*, and *E. hirae*, have been reported to suppress lung cancer angiogenesis. Further studies are needed for a thorough investigation into the potential role of bacteria influencing the metastatic process in lung cancer. 

Collectively, these findings indicate that the microbiota plays a crucial role in the development of lung cancer. Modulating the immune response and treating the local and distal microbiota represents a potential new avenue for lung cancer prevention and treatment.

## 5. Local Microbiota Role in Lung Cancer 

**Figure 2:**
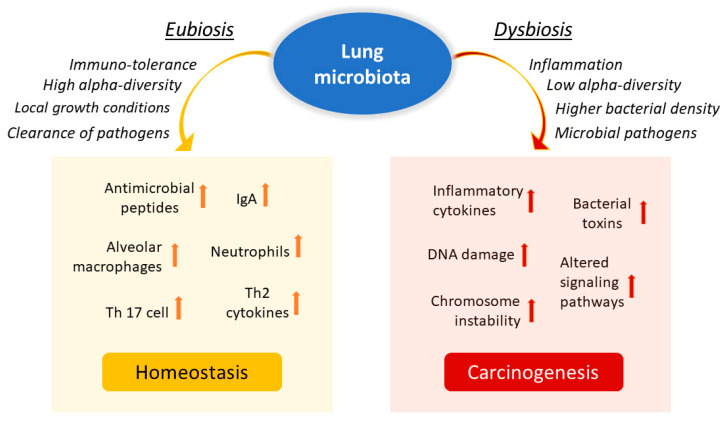
Influence of lung microbiota on homeostasis maintenance or carcinogenesis induction. The lung microbiota is crucial in driving local inflammation and tumor promotion. While the condition of eubiosis promotes immune tolerance and the formation of a homeostatic environment, dysbiosis and chronic infection of the lungs can cause alterations in the inflammatory response and result in a more hypoxic, tumor-promoting environment. Reduced alpha diversity and increased bacterial density are associated with lung cancer by stimulating the production of specific cytokines that promote inflammation (e.g., IL-17, IL-22) and the infiltration of neutrophils and other effector molecules that enhance the proliferation of cancer cells. Furthermore, enrichment of potential pathogenic bacterial taxa may facilitate changes in oncogenic pathways, possibly through some microbial toxins.

The lung is a critical site of immune–microbiota interaction, and homeostasis is maintained by the immune cells resident in the lung. Growing evidence from human and mouse studies has linked bacterial dysbiosis to lung cancer [12]. Many studies have observed reduced alpha diversity, enrichment in specific bacterial taxa, and higher bacterial density associated with lung cancer [78]. Chronic infection of the lungs can be the initial cause of cancer when microbiota dysbiosis results in a more hypoxic, tumor-promoting environment. In addition, anaerobic respiration is observed to increase in lung cancer due to the elective anaerobic qualities of the bacteria that preferentially colonize tumors. They grow in number along with cancer progression, further promoting and stabilizing the hypoxic and proinflammatory tumor environment [66]. Jin and collaborators showed that lung tumor burden was highly correlated with local bacterial abundance in the airways, but not in the intestinal tract, and deduced a relatively more significant contribution of the local microbiota in lung cancer development. Furthermore, they showed that intratracheal inoculation of a pool of bacteria isolated from advanced lung tumors significantly accelerated tumor growth [79] (Figure 2). 

While recent literature has focused primarily on the gut microbiota, it is unclear how the distal gut microbiota and the local microbiota work together to regulate the balance between tumor-promoting inflammation and antitumor immunity.

A decrease in the alpha diversity of the bacterial community in tumor tissues compared to non-malignant lung tissues has been reported in patients with lung cancer, while the beta diversity does not vary between healthy and malignant lung tissue [77]. Although there are no shared definitions for healthy or harmful lung microbiota, interesting correlations between specific taxa or genera and lung cancer have been identified by several authors. Jin and coworkers demonstrated the importance of microbiota-immunity cross-talk in promoting inflammation and lung cancer development in an indigenous mouse model [79]. Specifically, they found that some bacterial families, such as *Herbaspirillum* and *Sphingomonadaceae* were enriched in tumor-bearing lung tissues compared to healthy lungs. In contrast, other taxa, including *Aggregatibacter* and *Lactobacillus*, were enriched in healthy lungs. The increase in local bacterial load and the altered composition of the lung microbiota stimulated the production of IL-17, promoting inflammation and infiltration of neutrophils and IL-22 and other effector molecules that promote the proliferation of cancer cells. Germ-free mice or antibiotic-treated mice had significantly reduced lung tumor growth, which agrees with pulmonary physiology, providing an objective low microbial load. Yu and coworkers [80] observed an increase in the genera *Thermus* and *Legionella pneumophila* in patients with advanced lung cancer (IIIB, IV) and with metastases, respectively.

Using diagnostic bronchoscopy samples, Tsay and coworkers [81] found that lung cancer patients had increased oral taxa, particularly *Streptococcus* and *Veillonella*, compared to controls. The increased prevalence of oral taxa has been associated with PI3K and ERK (MAPkinase pathway) upregulation. In vitro experiments that exposed airway epithelial cells to *Veillonella, Prevotella, and Streptococcus* also led to the upregulation of the ERK and PI3K pathway, this last being implicated as an early event in lung carcinogenesis [82], and thus upregulation of this pathway by commensal microbiota dysbiosis facilitates carcinogenesis. Still, the results of Greathouse and coworkers [78] have suggested an association between TP53 and lung microbiota dysbiosis. The genus Acidovorax was enriched in squamous cell carcinoma lung biopsy specimens, and it was found that this same taxon is further enriched in lung biopsies of patients with TP53-mutated squamous cell carcinoma. In the lung cancer-associated microbiota, a significant enrichment of *Firmicutes Granulicatella*, *Abiotrophia*, and *Streptococcus* and a decrease in bacterial community diversity have been evidenced [83]. Moreover, the genus *Thermus* is more abundant in the tissues of advanced-stage cancer patients, while the presence of *Legionella* is more consistent in people who develop metastases [12]. Liu and coworkers provided evidence that the lung cancer-associated microbiota is enriched in *Streptococcus* while it is deficient in *Staphylococcus* [84], suggesting a deleterious role for the former and a protective role for the latter in lung cancer development [17]. In contrast, other researchers showed that *Staphylococcus* might induce DNA damage while *Streptococcus* may play a role in cancer prevention [85]. Such contradictory results, however, could be explained by the difficulties in identifying actual species or strains involved in carcinogenesis, or by the fact that different taxa could play distinct roles in different body niches or that even the same taxa may have protective or harmful functions in the same place, depending on the presence of other stimuli. It should be considered that the composition of the lung microbiota is associated with lifestyle, pollution, and tobacco smoke. Differences are also observable between patients with chronic bronchitis or tumors. It is worth noting that smokers’ intratumoral and intracellular bacterial taxa show enrichment in the chemical breakdown pathways of cigarette smoke, indicating an association between intratumoral microbiota and cancer etiology [45]. Hence, it appears crucial to consider the microbiota within the microenvironment in which it develops to favor its balance. 

Also, dysregulation of lung microbial communities probably facilitates changes in oncogenic pathways, potentially through specific microbial components such as toxins [12,86]. For instance, Apopa and collaborators [87] found an abundance of *Cyanobacteria* in NSCLC biopsies and related the microcystin toxin produced by the *Cyanobacteria* themselves with the local inflammatory state and the onset of lung cancer. Conversely, Yaghoobi and collaborators [88] have shown the anticancer properties of the CDT secreted by some pathogenic gram-negative bacteria. CDT induced apoptosis in the A549 human lung adenocarcinoma cell line, and potential antitumor use as a drug against lung cancer was speculated for the toxin. Intriguingly, CDT has also been indicated, among others, as a pro-carcinogenic toxin in the colon [68]. This apparent discrepancy, with the same microbial factor playing opposite roles in different environments, is not a new finding and certainly deserves a deeper investigation.

These findings strongly suggest the importance of the local microbiota in driving local inflammation and tumor promotion.

## 6. Elective Therapy in Lung Cancer and the Role of the Microbiota

It is increasingly recognized that the gut microbiota modulates the host response to chemotherapy drugs, with three main clinical outcomes: facilitating drug efficacy, cancellation, and impairment of the anticancer effects, mediation of toxicity. The implication is that the gut microbiota is critical for the development of personalized cancer treatment strategies, even for districts other than the intestine. Therefore, a greater understanding of the prokaryotic co-metabolism of chemotherapy drugs is urgently needed. This thinking is based on the evidence from human, animal, and in vitro studies that intestinal bacteria are strictly related to the pharmacological effects of chemotherapies and can consequently represent a target to improve efficacy and reduce the toxicity of current chemotherapeutic agents. Dysbiosis appears to be the consequence and often the reason for the variance observed in responses to therapy. More and more studies show that an intact microbiome is necessary for effective tumor control in response to genotoxic and immunomodulatory therapies [89].

In fact, a good balance between the gut microbiota and the immune system is a decisive key to maintaining the effectiveness of anticancer chemotherapy [90]. Elimination of the microbiota by administering broad-spectrum antibiotics alters host response, since genes that promote cancer metabolism and progression are upregulated, with simultaneous downregulation in inflammatory, phagocytic, and antigen presentation pathways [91]. It is evident that chemotherapy is dependent on the composition of the microbiota of patients, although the impact on prognosis is still unclear.

Regarding immunotherapy, numerous studies have already shown that the gut microbiota has the potential to stimulate the antitumor immune response. Monoclonal antibodies directed against CTLA-4 (ipilimumab), PD-1 (nivolumab), and PDL1 (pembrolizumab) are immune checkpoint inhibitors (ICIs) that induce a patient’s individual immune response against a tumor. These monoclonal antibodies appear to be highly effective in treating different types of cancer (melanoma, Hodgkin’s lymphoma, lung, kidney, bladder cancer, etc.). The effectiveness of ICIs appears to depend on the patient’s gut microbiota, which in turn interacts closely with the patient’s immune system. Therefore, this interaction between gut microbiota and ICIs may explain the interindividual variation reported in patient responses to immunotherapy [92].

## 7. Probiotic Therapies and Next-Generation Probiotics (NGPs)

A probiotic is currently indicated as a specific bacterial strain that can effectively promote human health and that should not carry or transfer antibiotic resistance to other strains in the microbiota. Although probiotics do not need to colonize the target organ, such as the intestine, at least a certain number of bacteria must reach the colon, where they can experience the local intestinal ecology, physiology, and metabolism [93]. By definition, probiotics should be safe in animals, resistant to acidity and bile acids, and able to adhere and colonize in the intestine. 

Available data indicate that probiotic bacteria can modulate the immune system by promoting the host’s endogenous defense systems and can modify various immune parameters, including humoral, cellular, and non-specific immunity. Emerging data also indicate that probiotics potentiate NK cell activity in the elderly and modulate non-specific host defenses. A reversal of the age-related decrease in cytokine production was demonstrated in elderly mice fed probiotic supplements. Immunomodulatory mechanisms that have been demonstrated with probiotics include induction of mucus production, activation of macrophages by *Lactobacilli* signaling, stimulation of secretory IgA and neutrophils, inhibition of the release of inflammatory cytokines, and stimulation of elevated peripheral immunoglobulins. In addition, probiotics can modulate DC surface phenotype and cytokine release. Other bacteria seem to be involved in the control of inflammation, side effects and chemotherapy-related toxicity in cancer patients. A combination of selected strains of *Lactobacilli* and *Bifidobacteria* has proven significant efficacy in hematological toxicity, affecting red blood cell and platelet count, and an improvement in the control of AST, GGT, urea, and creatinine levels in mice affected by pancreas cancer treated with gemcitabine plus probiotics compared to mice receiving chemotherapy alone [94]. Clearly, further investigation is needed to characterize candidate probiotic bacteria immunomodulatory properties and tailor their application to specific target populations.

In general, widely used traditional probiotics, such as *Bifidobacterium* spp., *Lactobacillus* spp., and many others, have been selected by chance or through the collection of life experiences. Although most of them show biological safety and some may show ameliorative efficacy, the overall effects and functions on disease improvement are statistically marginal. On the other hand, the administration of traditional probiotics does not target specific diseases.

Through analyses using the latest generation sequencing and bioinformatics platforms, many potential NGPs are currently under intensive development. Emerging NGPs include a class of organisms developed exclusively for pharmaceutical applications and as novel preventive tools. These comprise, for example, *Prevotella copri* and *Christensenella minuta*, which control insulin resistance, *Parabacteroides goldsteinii*, *A. muciniphila*, and *Bacteroides thetaiotaomicron*, which reverse obesity and insulin resistance, *Faecalibacterium prausnitzii,* which protects mice from intestinal disease, and *B. fragilis*, which reduces inflammation and antitumor effect. Some other bacterial species have also shown a promising efficacy in promoting anticancer immunotherapies. These bacteria include *Eubacterium limosum*, *E. hirae*, *Enterococcus faecium*, *Collinsella aerofaciens*, and *Burkholderia cepacia*.

Many of the aforementioned bacteria (*A. muciniphila*, *F. prausnitzii*, *Bifidobacterium spp.*, and *B. fragilis*) are associated with good clinical responses to immunotherapy. In contrast, they have also been found in various disease groups and associated with pathology. For example, according to some studies, *A. muciniphila* is positively correlated with Parkinson’s disease and constipation-predominant irritable bowel syndrome [95,96]. Other authors evaluate the pathogenic potential of *A. muciniphila* for its ability to adhere to and degrade the intestinal mucus layer as a starter of early pathogenic behaviors. Unlike pathogens, however, *A. muciniphila* as a mucin-degrading agent remains mainly in the outer mucous layer and does not reach the inner mucous layer, and it has been shown that bacteria reaching the inner layer are necessary for pathogenicity. Although mucin degradation itself is pathogen-like behavior, it is considered a normal process in the intestinal self-renewal balance. Furthermore, it has been reported that *A. muciniphila* can maintain host–gut microbial balance by converting mucin into beneficial products [97]. Up to now, *A. muciniphila* alone has not been proved to cause pathogenicity, although it is not known whether it can cause diseases in synergy with other bacteria.

The underlying reason for the ambivalent correlations of such microorganisms may be that the abundance of these health-associated bacteria may reflect the presence of a well-balanced intestinal microflora, leading to a homeostatic host–microbiota ecosystem associated with good health. The other possibility is that a small number of these NGP candidates may be directly responsible for the aberrant “immune set point”. However, it still remains unclear whether a single bacterial strain is sufficient to obtain such ameliorative effects or whether cocktails are required to obtain the effects of live bacterial biotherapy [98].

## 8. Treatment of the Gut Microbiota in Lung Cancer

The gut microbiota characteristics in lung cancer patients vary widely compared to healthy subjects, suggesting its possible involvement in lung cancer prognosis and therapy. The gut microbiota may significantly influence immunotherapy by altering the differentiation of regulatory T cells, thus resulting in changes in immunomodulation mechanisms [99]. The same authors found that supplementation with *A. muciniphila* increases response to immunotherapy, while an abnormal gut microbiota composition is associated with resistance to the treatment [99]. The gut microbiota in lung cancer patients who respond to immunotherapy differs significantly from that of unresponsive patients. Furthermore, a significantly higher response to anti-PD-1 therapy in lung cancer patients was found to be positively correlated with *A. muciniphila* species abundance [17].

Recent work has shown that gut microbiota diversity in fecal bacteria (*Proteobacteria*, *Firmicutes*, *Bacteroidetes*, and *Actinobacteria*) increases the response to anti-PD-1 immunotherapy [100]. Additionally, a previous study stated that in patients with NSCLCs that responded to nivolumab, the gut microbiota composition was relatively stable, and greater diversity was noted. Extended progression-free survival has also been demonstrated in patients with high microbiome diversity versus those with low diversity. Analysis of systemic immune responses by multicolor flow cytometry revealed that patients with high gut microbiome diversity have a higher frequency of CD8 + T cells with unique memory and subsets of NK cells in the periphery in response to anti-PD-1 [79]. A retrospective evaluation study of 118 patients with advanced NSCLC and treated with immunotherapy showed that the addition of supplemental therapy with *Clostridium butyricum* before and/or after immunotherapy resulted in significantly prolonged progression-free survival and overall survival of patients [101].

In addition to the observed improvements in response to immunotherapy, the gut microbiota also affects the efficacy of chemotherapy treatment in lung cancer. For example, the oral administration of *Lactobacillus acidophilus* during cisplatin treatment in mouse models of lung cancer has improved the anticancer efficacy of cisplatin, reduced tumor size, and improved survival rate. These results suggest that co-administration of probiotics enhances the antigrowth and pro-apoptotic effects of cisplatin [102]. In addition, patients with end-stage lung cancer and undergoing chemo-immunotherapy, who additionally received *E. hirae* and *Barnesiella intestinihominis,* had longer progression-free survival [103]. Therefore, the increased survival of these patients can be attributed to the improvement of the immunomodulatory effect.

A study by Liu et al. [104], in which the gut microbiota of 30 lung cancer patients and 16 healthy subjects was analyzed, found that each lung cancer group had a loss of bacterial diversity and a shortage of the probiotic genera *Blautia*, *Coprococcus*, *Bifidobacterium*, and *Lachnospiraceae*, compared to healthy controls. The genus *Blautia*, belonging to the *Firmicutes* phylum, has the role of helping to digest complex carbohydrates. Decreases in the *Blautia* genus have also been observed in irritable bowel syndrome, non-alcoholic fatty liver disease, Crohn’s disease, and diabetes. *Coprococcus*, observed only in the control group, is a beneficial butyrate-producing genus. Other studies have shown that high consumption of yogurt is beneficial as it has been shown to cause a significant reduction in lung cancer risk by 30%, implying that prebiotics and probiotics may have a protective effect on lung carcinogenesis [105]. This evidence indicates that the composition and development of bacterial communities vary in lung cancer with different biomarkers. Therefore, it is possible that some special microbiomes may serve for diagnosis, prognosis, therapeutic target or for fecal microbiota transplantation in lung cancer therapy.

However, the role of the gut microbiota in the development and progression of lung cancer needs further investigation, and the potential actions of the microbiome in the effective modulation of anticancer treatment should be further explored and evaluated.

## 9. Conclusions

The gut microbiota is largely indicated as a key player in tumor treatment; it has been shown to be directly involved in tumor biology, such as the transformation process, tumor progression, and response to anticancer therapies, including immunotherapy. Characterization of the intratumoral microbiota may also be an essential step in unraveling the effects that bacteria may have on the different hallmarks of cancer.

Whether or not bacteria play a causal role in tumorigenesis, it is interesting to explore further the effects that bacteria may have on different tumor cell phenotypes and on the immune system, and its interactions with cancer cells. The role of the commensal microbiota in tumorigenesis has been more thoroughly investigated for those tumors associated with mucous membranes that interface with the external environment, as in the case of the lung. For these, in fact, the correlations appear to be more direct and the analytical challenges more easily sustainable. It should also be considered that lung cancer is one of the deadliest cancers in the world [60], and NSCLC accounts for the majority of lung cancer cases [106]. Therefore, the mechanisms by which the microbiota can influence cancer progression deserve deeper attention to improve patient survival and responses to treatment. Although many authors strongly suggest the importance of the local versus the distal (gut) microbiota in driving local inflammation and tumor promotion, the role of the gut microbiota in lung tumorigenesis remains relatively unexplored. However, it is agreed that gut microbiota characteristics in lung cancer patients vary widely compared to healthy subjects. It is also shown that the intestinal microbiota in lung cancer patients responding to immunotherapy treatment differ significantly from that of patients who do not respond to immunotherapy. All in all, these studies suggest that the gut microbiota may influence lung cancer prognosis and therapy.

However, emerging experimental and epidemiological evidence points to the GLA, a vital dialog between the mucosal tissues of our body that causes changes in the gut microenvironment, impacts on both health and disease, and could be profoundly important for both the etiology and treatment of diseases in other districts. In fact, specific intestinal probiotic strains also show promise in the treatment of airway diseases. Several studies have evaluated the correlations between the relative abundance of specific bacterial genera and the systemic blood markers related to inflammation, eventually identifying phyla or genera with probiotic characteristics, and single species associated with improved therapeutic responses and disease-free survival. In view of this goal, research is significantly focused on the development of NGPs, for which their speciality is precisely the expression of well-defined and targeted functions used as tools to design personalized medicines.

Characterization of individual bacteria has sometimes led to contradictory results and ambiguous prognostic correlations. This suggests we should not only focus on the specific functions of single bacteria, but also consider the surrounding microbiota composition, eubiotic or dysbiotic status, and the influence it may exert in selecting specific bacterial functions, within a very complex universe of communications that involves the entire host. In contrast, data that agree with all the observations of favorable correlations concern the importance of biodiversity in intestinal microbiota, where a greater variety of bacterial families is solidly correlated with better tumor prognosis, better therapeutic responses, and disease-free survival. For the lung microbiota, even greater biodiversity and lower bacterial density are associated with a health condition. 

Restoring the homeostasis and eubiosis of the bacterial microbiota as a whole should be the basic objective of all interventions on the microbiota. Although many of the abovementioned probiotics are not yet available for humans, promoting a correct lifestyle and diet is a daily practice that can influence microbiota composition. Selecting specific prebiotics, probiotics, and integrative postbiotics is a personalized and tailored approach to support host health.

## Data Availability

The study did not report any original data.

## Abbreviations

AMPs	antimicrobial peptides
BM	brain metastasis
CDT	cytolethal distending toxin
CNF1	cytotoxic necrotizing factor 1
DCs	dendritic cells
DM	distant metastasis
EMT	Epithelial–mesenchymal transition
GLA	gut–lung axis
GPCRs	G protein-coupled receptors
ICIs	immune checkpoint inhibitors
MAITs	mucosal-associated invariant T cells
MIS	mucosal immune system
NGPs	next-generation probiotics
NK	natural killer
NSCLCs	non-small-cell lung cancers
PCs	plasma cells
PPs	Peyer’s patches
SCFAs	short-chain fatty acids
TLR	toll-like receptor
TNM	tumor-node-metastasis stage
